# Dr. Ludolph Parmly

**Published:** 1854-07

**Authors:** 


Obituary.—We learn with profound sorrow that Dr. Ludolph Parmly, Dentist, of Mobile, Ala., died during the latter part of the present month (July.) Dr. P. had resided in Mobile about twenty-two or twenty-three years, and during which time, he enjoyed, and deservedly too, a large and lucrative practice. In his death, the profession and society have lost one of their brightest ornaments, and most sincerely do we sympathize with his family and numerous relatives in their bereavement. Dr. P. was an honorable, a high and a liberal minded gentleman, as well as a scrupulously conscientious and eminently skillful practitioner.

